# Secular trends and social inequalities in child behavioural problems across three Brazilian cohort studies (1993, 2004 and 2015)

**DOI:** 10.1017/S2045796023000185

**Published:** 2023-04-17

**Authors:** Michelle Degli Esposti, Alicia Matijasevich, Stephan Collishaw, Thaís Martins-Silva, Iná S. Santos, Ana Maria Baptista Menezes, Marlos Rodrigues Domingues, Fernando C. Wehrmeister, Fernando Barros, Joseph Murray

**Affiliations:** 1 Human Development and Violence Research Centre (DOVE), Federal University of Pelotas, Pelotas, Brazil; 2 Department of Social Policy and Intervention, University of Oxford, Oxford, UK; 3 Departamento de Medicina Preventiva, Faculdade de Medicina FMUSP, University of São Paulo, São Paulo, Brazil; 4 Wolfson Centre for Young People’s Mental Health and MRC Centre for Neuropsychiatric Genetics and Genomics, Division of Psychological Medicine and Clinical Neurosciences, School of Medicine, Cardiff University, Cardiff, Wales; 5 Postgraduate Program in Epidemiology, Federal University of Pelotas, Pelotas, Brazil; 6 Post Graduate Course in Health in the Life Cycle, Universidade Católica de Pelotas, Pelotas, Brazil

**Keywords:** behaviour problems, epidemiology, population survey, social factors, time trends

## Abstract

**Aims:**

Previous epidemiological evidence identified a concerning increase in behavioural problems among young children from 1997 to 2008 in Brazil. However, it is unclear whether behavioural problems have continued to increase, if secular changes vary between sociodemographic groups and what might explain changes over time. We aimed to monitor changes in child behavioural problems over a 22-year period from 1997 to 2019, examine changing social inequalities and explore potential explanations for recent changes in behavioural problems between 2008 and 2019.

**Methods:**

The Child Behaviour Checklist was used to compare parent-reported behavioural problems in 4-year-old children across three Brazilian birth cohorts assessed in 1997 (1993 cohort, *n* = 633), 2008 (2004 cohort, *n* = 3750) and 2019 (2015 cohort, *n* = 577). Response rates across all three population-based cohorts were over 90%. Moderation analyses tested if cross-cohort changes differed by social inequalities (demographic and socioeconomic position), while explanatory models explored whether changes in hypothesized risk and protective factors in prenatal development (e.g., smoking during pregnancy) and family life (e.g., maternal depression and harsh parenting) accounted for changes in child behavioural problems from 2008 to 2019.

**Results:**

Initial increases in child behavioural problems from 1997 to 2008 were followed by declines in conduct problems (mean change = −2.75; 95% confidence interval [CI]: −3.56, −1.94; *P* < 0.001), aggression (mean change = −1.84; 95% CI: −2.51, −1.17; *P* < 0.001) and rule-breaking behaviour (mean change = −0.91; 95% CI: −1.13, −0.69 *P* < 0.001) from 2008 to 2019. Sex differences in rule-breaking behaviour diminished during this 22-year period, whereas socioeconomic inequalities in behavioural problems emerged in 2008 and then remained relatively stable. Consequently, children from poorer and less educated families had higher behavioural problems, compared to more socially advantaged children, in the two more recent cohorts. Changes in measured risk and protective factors partly explained the reduction in behavioural problems from 2008 to 2019.

**Conclusions:**

Following a rise in child behavioural problems, there was a subsequent reduction in behavioural problems from 2008 to 2019. However, social inequalities increased and remained high. Continued monitoring of behavioural problems by subgroups is critical for closing the gap between socially advantaged and disadvantaged children and achieving health equity for the next generation.

## Introduction

Mental health problems are a major global health issue, affecting 10% to 20% of children and adolescents worldwide (Belfer, 2008). Disruptive behaviour disorders characterized by behavioural problems (oppositional defiant disorder and conduct disorder) are the second most common mental health disorder and, in 2015, were estimated to affect 113 million children and adolescents (Polanczyk *et al*., 2015). Children with behavioural problems are at risk of a range of adverse long-term outcomes, including poor education, psychiatric and substance use disorders, violence and criminality (Erskine *et al*., 2016). In addition to the costs to individuals and families, behavioural problems have high societal costs and burden public services (criminal justice, health and social welfare) (Erskine *et al*., 2014; Rissanen *et al*., 2021; Rivenbark *et al*., 2018). The prevention of child behavioural problems is an urgent public health priority (National Research Council, 2009), especially in low- and middle-income countries where the burden is disproportionately large (Crijnen *et al*., 1997; Kieling *et al*., 2011). Developing an accurate understanding of trends in low- and middle-income countries is therefore needed to plan prevention at a global scale.

Comparisons of epidemiological surveys measuring child behavioural problems, namely conduct problems (lying, disobedience, aggression and bullying), show broadly similar time trends across high-income countries (Collishaw, 2015). Studies conducted in Europe and North America consistently found increases in behavioural problems among children and adolescences from the 1970s up until the late 1990s (Achenbach *et al*., 2003; Collishaw *et al*., 2004). Recent studies suggest that rates of child behavioural problems have been levelling off – and even declining – since the 2000s (Bor *et al*., 2014; Maughan *et al*., 2008; Sellers *et al*., 2015). These secular trends also share similarities with national trends in police- and victim-reported crime in high-income countries, where crime rates climbed to a peak in the mid-1990s and then steadily declined (the ‘crime drop’) (Van Dijk *et al*., 2012).

Despite convergent evidence from high-income countries, there is limited epidemiological evidence on trends in behavioural problems from low- and middle-income countries – where 90% of the world’s children live (Kieling *et al*., 2011). The very few studies available suggest that trends in high-income countries are unlikely to be universal (Collishaw, 2015). One Brazilian study identified increasing levels of behavioural problems and aggression among young children from 1997 to 2008, which were concentrated among socially disadvantaged children (Matijasevich *et al*., 2014). This increase in child behaviour problems paralleled national increases in violence during the same period in Brazil (Murray *et al*., 2013). It is thus important to track trends in behavioural problems beyond 2008 to determine whether Brazilian children are increasingly at risk.

Besides tracking overall population-level changes in child behavioural problems, it is critical to understand which groups are most affected. Social inequalities are a major public health concern (Marmot, 2015; Marmot and Bell, 2016), and there is evidence of mental health disparities by sex and social disadvantage, which emerge early in life (Campbell *et al*., 2021; Reiss, 2013). There are also recent concerns that mental health gaps are widening between socially advantaged and disadvantaged children (Collishaw *et al*., 2019; Collishaw and Sellers, 2020). Brazil has long-been characterized by deep social inequalities (Paim *et al*., 2011), and initial evidence suggests that socially disadvantaged children suffered larger increases in behavioural problems than socially advantaged children, up to 2008 (Matijasevich *et al*., 2014). Understanding social inequalities in child mental health, and how they are manifesting over time, can help to identify those most at risk.

It is also important to understand the factors driving secular changes in child behavioural problems. Previous evidence from Brazil has identified significant improvements in social and environmental determinants of health including maternal characteristics (older mothers and higher education), parenting behaviours (e.g., breastfeeding patterns) and family income and household assets (Bertoldi *et al*., 2019; Santos *et al*., 2019). Such reductions in known risk factors for child behavioural problems – and the bolstering of protective factors – would be expected to result in overall improvements in behavioural problems over time (Hill, 2002; Murray and Farrington, 2010). However, a prior study found deteriorations in child behaviour from 1997 to 2008 (Matijasevich *et al*., 2014). This finding may be due to other secular changes at play. For example, the same study noted more recent increases in the proportion of single mothers and higher rates of maternal psychiatric problems, which may be counteracting more distal societal improvements and placing children at higher risk of behavioural problems (Matijasevich *et al*., 2014). Determining whether secular changes in certain risk and protective factors explain trends in child behavioural problems is key to informing wider prevention efforts and anticipating future need.

In this study, we aimed to advance the epidemiological evidence on secular trends in child behavioural problems and improve understanding of the aetiology of population-level changes in a Brazilian population. First, we assessed whether child behavioural problems continued to increase in years since 2008, examining the trends across three comparable birth cohorts (1993, 2004 and 2015). Second, we investigated inequalities in child behavioural problems during this 22-year period. Specifically, we tested whether the gap between boys and girls, and between socially advantaged and disadvantaged children, has changed with time. Third, we explored potential explanations for recent changes in child behavioural problems by investigating changes in risk and protective factors.

## Methods

This cross-cohort epidemiological study compares child behavioural problems across three longitudinal Brazilian studies: the 1993, 2004 and 2015 Pelotas Birth Cohort Studies. In each cohort, child behavioural problems were assessed at age 4 years and compared through time (in 1997, 2008 and 2019, respectively). We further examined changes in behavioural problems by socioeconomic factors, and whether changes in hypothesised risk and protective factors explain changes in behavioural problems between the 2004 and 2015 cohorts – extending a prior study examining change in behaviour problems between the 1993 and 2004 cohorts (Matijasevich *et al*., 2014). We follow recommendations set out in the STROBE statement for reporting observational studies in epidemiology (von Elm *et al*., 2007) and pre-registered an analysis plan at the Open Science Framework (osf.io/ps45y).

### Research setting and samples

Pelotas is a Southern Brazilian city with nearly 342,000 inhabitants (Instituto Brasileiro de Geografia e Estatística, 2010). Over 90% of its population is urban and more than 99% of all birth deliveries take place in hospitals. Children born from 1 January to 31 December for the years 1993, 2004 and 2015, and their mothers, were the target population for the three birth cohort studies. Nonresponse rate at recruitment was below 1% for all three cohorts, resulting in the following sample sizes (livebirths): 1993 (*n* = 5249), 2004 (*n* = 4231) and 2015 (*n* = 4275). Data were collected using consistent methodology across the three cohorts. For the perinatal interview, mothers were interviewed soon after delivery using a structured questionnaire about demographic, socioeconomic, behaviour and biological characteristics; reproductive history; and healthcare utilisation. Follow-ups were conducted at several time points, with high follow-up rates (Figure S1). Further information about the studies are detailed elsewhere (Hallal *et al*., 2018; Santos *et al*., 2011; Victora *et al*., 2008).

The 2004 cohort measured behavioural problems in 4-year-olds for all the cohort’s children included in the follow-up (*n* = 3750, 88.6%), whereas comparable data were collected for sub-samples in the 1993 and 2015 cohorts. The sub-sample from the 2015 cohort is a random sub-sample of approximately 15% of the cohort population who were assessed at age 4 years (*n* = 577), whereas the sub-sample from the 1993 cohort included all low birthweight children plus a random sample of 20% of the rest of the cohort assessed at age 4 years. Of the 1460 children in the 1993 cohort eligible for a wide range of measures at age 4 years, 87.2% were located and approximately half of those (*n* = 633) were selected and assessed for behavioural problems, oversampling from low birthweight children (see Missing data section; Matijasevich *et al*., 2014). We conducted power calculations to check whether sample sizes were powered to detect differences in child behavioural problems across cohorts (Appendix S1 and Figures S1–S3). Mean (standard deviation [SD]) ages, in months, at the age 4-year assessment for those with valid data on behavioural problems were 53.6 (3.67), 50.3 (1.79) and 45.0 (2.52), in the 1993, 2004 and 2015 cohorts, respectively.

### Measures

#### Child behavioural problems (4 years)

Child behavioural problems were assessed using the 4- to 18-year-old parent report version Child Behaviour Checklist (CBCL) in the 1993, 2004 and 2015 cohorts (Achenbach and Edelbrock, 1991). The CBCL has been previously validated for use among Brazilian children with good psychometric properties, including reliability and validity, in both clinical and non-clinical samples (Bordin *et al*., 1995). The 118 behavioural and emotional items of the CBCL were scored by the mothers, or other caregivers, and collected when the child was 4 years old. Of the eight empirically derived scales in the CBCL, we used two that measure the broad dimension of conduct problems: aggressive behaviour and rule-breaking behaviour. We analysed each dimension separately, as well as a summed score reflecting total conduct problems. All outcomes were modelled as continuous variables.

#### Demographic and socioeconomic position (birth)

We used measures of demographic and socioeconomic position that were consistently collected across cohorts during the perinatal interview (Table S1). We used demographic information on child sex (male; female), and maternal ethnicity, referring to mother’s skin colour (White, Black/mixed). We also used two measures of socioeconomic position (Barros and Victora, 2013; Howe *et al*., 2012): family income and maternal education. Family income was expressed in quintiles with the first quintile representing the poorest and the fifth quintile representing the richest families, while maternal education was measured by the number of years of formal schooling (0–4, 5–8 and ≥ 9 years).

#### Additional risk and protective factors

To investigate the potential explanations for changes in child behavioural problems between the 2004 and 2015 cohorts, we used demographic and socioeconomic position measures (described above) and additional information on prenatal and developmental factors, family structure, and maternal mental health and parenting. We selected these variables based on the following criteria: (i) risk or protective factors for behavioural problems based on prior theory and literature (Hill, 2002; Murray and Farrington, 2010), organised in a directed acyclic graph (DAG, see Figure S4); (ii) evidence and/or theoretical plausibility of change over time in Brazil (e.g., breastfeeding patterns) (Santos *et al*., 2019); and (iii) consistently measured across both the 2004 and 2015 cohorts (Hallal et al., 2018; Santos et al., 2011).

##### Prenatal and developmental factors

Measures included maternal smoking during pregnancy, maternal employment during pregnancy, gestational age (preterm births < 37 weeks of gestation), child’s birth weight (low birthweight < 2500 g), breastfeeding at 12 months and neurocognitive development (suspected delay; normal). Information on whether children were still breastfed at 12 months old were collected during the 12-month follow-up interview, and screening for neurodevelopmental delay was assessed using Battelle’s Development Inventory (BDI) when the children were 4 years old. We used previously validated cut-offs for BDI where children scoring 1.5 standard deviations below the mean of the cohort were defined as having suspected developmental delay (Bertoldi *et al*., 2019; de Moura *et al*., 2010; Elbaum *et al*., 2010; Santos *et al*., 2019). All other measures were collected during the perinatal interview.

##### Family structure

Maternal age (≤19, 20–34, ≥ 35 years) and parity (0, 1, ≥ 2 child births) were measured during the perinatal interview, while mother’s marital status (with partner, single) was measured at 12-month follow-up.

##### Maternal mental health and parenting

Depressive symptoms among mothers were measured using the Edinburgh Postnatal Depression Scale when their child was 12 and 3 months old for the 2004 and 2015 cohorts, respectively. In line with previous literature, we applied a ≥10 cut-off point to indicate the presence of depressive symptoms (Santos *et al*., 2007). Harsh parenting was measured using interviewer ratings of parent–child interactions during their home visits in the 2004 cohort and during research centre visits in the 2015 cohort when the children were aged 4 years old, which was correlated (*P* < 0.05) with previously validated measures (e.g., Conflict Tactics Scale). We defined harsh parenting (binary: yes/no) if the interviewer indicated that the mother showed two or more of the following behaviours towards the child: (i) lack of affection or praise; (ii) indifference; (iii) threatened or scolded; and (iv) hit the child during the interview. At the 4-year follow-up, five activities relating to child stimulation were recorded (each item a binary variable; yes/no): in the last week, someone read/told a story to the child; the child went to a park/playground; went to other people’s houses; watched TV and the child had a story book at home. As in previous work, positive answers were summed to form a total score ranging from 0 to 5 indicating overall child stimulation (Barros *et al*., 2010).

### Analyses

We examined whether mean levels of child behavioural problems changed across the three cohorts, while adjusting for child’s age in months at 4-year assessment. Regression analyses modelled study cohort as independent categorical variables to test for cross-cohort changes in outcomes. Conduct problems, aggressive and rule-breaking behaviours were analysed separately throughout all analyses.

Next, we tested for differences in child behavioural problems by demographic and socioeconomic position measures both within and across cohorts. We collapsed family income into two categories representing poorer (bottom two quintiles) and richer children (top three quintiles), and maternal education into two categories representing children with less (0–8 schooling years) and more educated mothers (≥9 years) to ensure statistical power (see Appendix S1 and Figures S2–S3 for power calculation details motivating this). We also calculated inequality indices – the slope index of inequality and relative index of inequality) – which model differences in outcome across the whole distribution of family income and maternal education (Barros and Victora, 2013) (see Appendix S2). To investigate whether cross-cohort changes child behavioural problems were moderated by demographic and socioeconomic position measures, we modelled an interaction term between study cohort (categorical) and each socioeconomic factor.

We also investigated whether changes in child behavioural problems between the 2004 and 2015 cohorts are explained by changes in demographics, socioeconomic position, prenatal and developmental factors, family structure, and maternal mental health and parenting. First, we checked for differences using Pearson chi-squared (χ^2^) tests. Second, we investigated their associations with child behavioural problems, using the Benjamini–Hochberg method to control for the false discovery rate (Benjamini and Hochberg, 1995). Then, we assessed the impact of controlling for changes in risk and protective factors on estimates of changes in behavioural problems. Third, we iteratively fitted a series of adjusted models before a final fully adjusted model (Figure S4), applying backward elimination according to Akaike Information Criterion to identify and retain important explanatory variables (Heinze *et al*., 2018). Comparing unadjusted and adjusted estimates indicates whether changes in these factors contributed to recent changes in behavioural problems, in line with previous methodology (Collishaw *et al*., 2012), as visualised in Figure S5.

#### Missing data

Given the nature of the 1993 sub-sample, we used probability weights of 0.33 for low birthweight children and 1.28 for the rest of the sample to statistically adjust for oversampling and to match all the cohort’s children, as in previous work (Barros *et al*., 2012; Matijasevich *et al*., 2014). We present the weighted results for complete cases throughout the manuscript; unweighted are available on request.

## Results

### Changes in child behavioural problems through time

**Fig. 1.**
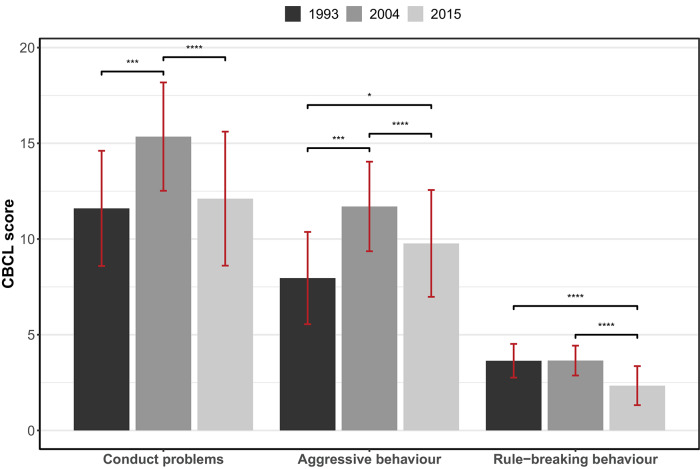
Child behavioural problems across the 1993, 2004 and 2015 cohorts.

Figure 1 shows child behavioural problem scores across the 1993, 2004 and 2015 cohorts. Total conduct problems and aggressive behaviour increased between the 1993 and 2004 cohorts, while there was no change in rule-breaking behaviours. All three behavioural outcomes, however, decreased from the 2004 to 2015 cohorts (Table 1 and Table S2).

Bars represent mean CBCL scores with standard error of mean (red) with *P* values derived from cross-cohort comparisons adjusted for age (months) at time of testing. *P < .05; **P < .01; ***P < .001.

**Table 1.** Cross-cohort change in child behavioural problems moderated by child sex and socioeconomic position

	Cohort comparisons, *b* [95% CI]					
	Conduct problems		Aggressive behaviour		Rule-breaking behaviour	
	1993 vs 2004 cohort	2004 vs 2015 cohort	1993 vs 2004 cohort	2004 vs 2015 cohort	1993 vs 2004 cohort	2004 vs 2015 cohort
**Total sample**	3.28 [2.5, 4.06]***	−2.75 [−3.56, −1.94]***	3.22 [2.58, 3.85]***	−1.84 [−2.51, −1.17]***	0.06 [−0.16, 0.28]	−0.91 [−1.13, −0.69]***
**Child’s sex**						
Male	3.03 [1.98, 4.08]***	−3.05 [−4.03, −2.07]***	3.07 [2.22, 3.93]***	−2 [−2.82, −1.18]***	−0.04 [−0.36, 0.27]	−1.05 [−1.32, −0.79]***
Female	3.5 [2.42, 4.58]***	−2.44 [−3.42, −1.45]***	3.35 [2.47, 4.23]***	−1.68 [−2.5, −0.86]***	0.15 [−0.14, 0.43]	−0.76 [−1.02, −0.49]***
Interaction effect	0.47 [−1, 1.93]	0.61 [−0.52, 1.74]	0.28 [−0.91, 1.47]	0.32 [−0.63, 1.26]	0.19 [−0.22, 0.61]	0.29 [0, 0.58]*
**Ethnic background (mother’s skin color)**						
White	2.79 [1.94, 3.64]***	−2.63 [−3.49, −1.77]***	2.88 [2.18, 3.58]***	−1.77 [−2.49, −1.05]***	−0.09 [−0.33, 0.15]	−0.86 [−1.09, −0.64]***
Black/mixed	4.6 [2.97, 6.23]***	−3.31 [−4.57, −2.05]***	4.11 [2.8, 5.42]***	−2.22 [−3.24, −1.19]***	0.49 [0.05, 0.94]*	−1.1 [−1.45, −0.75]***
Interaction effect	1.82 [0.03, 3.6]*	−0.68 [−1.96, 0.6]	1.23 [−0.2, 2.67]	−0.45 [−1.5, 0.61]	0.58 [0.09, 1.08]*	−0.23 [−0.58, 0.11]
**Family income**						
Richer (Q3–Q5)	2.33 [1.33, 3.33]***	−2.58 [−3.43, −1.72]***	2.56 [1.73, 3.38]***	−1.81 [−2.53, −1.09]***	−0.23 [−0.5, 0.05]	−0.77 [−1, −0.54]***
Poorer (Q1–Q2)	4.73 [3.54, 5.93]***	−3.07 [−4.22, −1.92]***	4.24 [3.28, 5.2]***	−1.93 [−2.88, −0.98]***	0.5 [0.14, 0.85]**	−1.13 [−1.45, −0.82]***
Interaction effect	2.4 [0.88, 3.93]**	−0.49 [−1.68, 0.69]	1.68 [0.46, 2.91]**	−0.13 [−1.11, 0.86]	0.72 [0.28, 1.16]**	−0.36 [−0.67, −0.06]*
**Maternal education (schooling years)**						
Higher (≥9 y)	1.69 [0.41, 2.98]**	−1.73 [−2.59, −0.87]***	2.25 [1.18, 3.31]***	−1.14 [−1.86, −0.42]**	−0.56 [−0.92, −0.19]**	−0.59 [−0.82, −0.36]***
Lower (0–8 y)	4.37 [3.44, 5.3]***	−3.19 [−4.41, −1.97]***	3.91 [3.15, 4.66]***	−2.1 [−3.11, −1.09]***	0.46 [0.2, 0.72]***	−1.09 [−1.41, −0.78]***
Interaction effect	2.68 [1.14, 4.21]***	−1.47 [−2.72, −0.21]*	1.66 [0.39, 2.93]*	−0.96 [−2, 0.08]	1.02 [0.58, 1.45]***	−0.5 [−0.82, −0.18]**

Unstandardised regression coefficients (*b*) represent change in CBCL scores between cohorts, adjusted for age (months) at time of testing. Moderation of cross-cohort change was tested by modelling interaction effects of study cohort × social inequality measure. *b*, beta coefficient; CI, confidence interval; Q, quintiles; y, years.

* *P* < .05,

** *P* < .01,

*** *P* < .001.

### Changes in child behavioural problems by demographic and socioeconomic factors

**Fig. 2.**
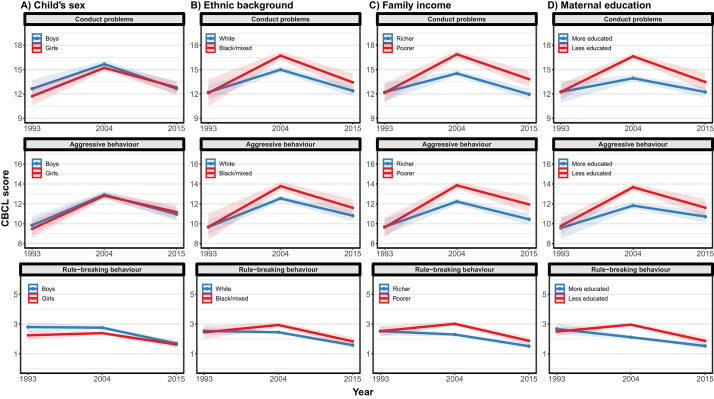
Changes in child behavioural problems by demographic and socioeconomic factors across the 1993, 2004 and 2015 cohorts.

Social inequalities (demographic and socioeconomic differences) in child behavioural problems were first examined within each cohort (Table S3). Girls had lower levels of rule-breaking behaviour than boys in the 1993 and 2004 cohorts. Socially disadvantaged children (poorer and lower maternal education) had higher behavioural problems than more advantaged children in the 2004 and 2015 cohorts (Tables S4 and S5). Next, we examined whether change in behavioural problems was moderated by demographic and socioeconomic position via interaction effects (Table 1 and Figure 2). The recent reductions in rule-breaking behaviour were moderated by the child’s sex, where decreases in rule-breaking behaviour were more pronounced among boys than girls (interaction *P* = 0.047). Children of Black or mixed mothers saw larger increases in conduct problems and aggressive behaviour between the 1993 and 2004 cohorts (interactions: *P* = 0.046 and *P* = 0.093, respectively), but the reduction between the two more recent cohorts was not moderated by ethnic background.

Lines represent estimated cross-cohort changes in CBCL scores by demographic and socioeconomic factors and their corresponding 95% confidence intervals, while adjusting for child’s age (months at time of testing).

Socioeconomic position, measured by both family income and maternal education, moderated the changes in behavioural problems between the 1993 and 2004 cohorts (Table 1 and Figure 2). The increases in conduct problems and aggressive behaviour among children from poorer families were approximately double the increases seen among children from richer families (interactions *P* = 0.002 and *P* = 0.007, respectively). Children of less educated mothers showed even more pronounced increases in conduct problems and aggressive behaviour between the 1993 and 2004 cohorts. Socially disadvantaged children, however, did show larger reductions in rule-breaking behaviour between the 2004 and 2015 cohorts.

### Explaining reductions in child behavioural between the 2004 problems and 2015 cohort

Compared to the 2004 cohort, fewer mothers in the 2015 cohort smoked during pregnancy (27.2% vs 15.3%) and were depressed (27.2% vs 18.1%). There was also a higher proportion of mothers who were older (e.g., 35 years+: 13.7% vs 20.4%), received at least 9 years of education (43.2% vs 66.2%) and were employed during pregnancy (40.7% vs 56.3%). Although children engaged in more stimulating activities, there was more evidence of harsh parenting in the 2015 cohort (Table S6). In addition to family income and maternal education, smoking during pregnancy and harsh parenting were consistently associated with higher behavioural problems (Table 2). Other maternal characteristics and parenting behaviours were also significantly correlated with a higher risk of child behavioural problems in the 2004, but not the 2015 cohort. These included younger motherhood, single marital status, more depressive symptoms and engaging their child in fewer child socially and cognitively stimulating activities (see Table 2).

**Table 2.** Unadjusted associations between risk and protective factors and child behavioural problems in the 2004 and 2015 cohorts

	Conduct problems		Aggressive behaviour		Rule-breaking behaviour	
	2004 Cohort	2015 Cohort	2004 Cohort	2015 Cohort	2004 Cohort	2015 Cohort
Child’s age at time of testing	0.01 [−0.12, 0.13]	−0.01 [−0.21, 0.2]	0.03 [−0.07, 0.14]	0 [−0.17, 0.17]	−0.03 [−0.06, 0.01]	0 [−0.06, 0.05]
**Demographic and socioeconomic factors**						
Child’s sex	−0.46 [−0.91, 0]	0.16 [−0.88, 1.2]	−0.09 [−0.46, 0.28]	0.24 [−0.63, 1.11]	−0.36 [−0.5, −0.23]***	−0.08 [−0.34, 0.18]
Ethnic background (mother’s skin colour)	1.73 [1.22, 2.24]***	1.05 [−0.07, 2.17]	1.24 [0.82, 1.66]***	0.8 [−0.14, 1.74]	0.49 [0.34, 0.64]***	0.25 [−0.03, 0.53]
Family income (quintiles)	−0.99 [−1.15, −0.83]***	−0.73 [−1.1, −0.35]***	−0.7 [−0.83, −0.57]***	−0.58 [−0.89, −0.26]***	−0.28 [−0.33, −0.24]***	−0.15 [−0.25, −0.06]**
Maternal education (schooling years)	−1.8 [−2.12, −1.48] ***	−1.08 [−1.9, −0.26]*	−1.24 [−1.5, −0.97]***	−0.86 [−1.54, −0.17]*	−0.56 [−0.66, − 0.47]***	−0.23 [−0.43, −0.02]
**Prenatal and developmental factors**						
Maternal smoking during pregnancy	3.3 [2.8, 3.81]***	2.19 [0.76, 3.61]**	2.41 [2, 2.82]***	1.86 [0.67, 3.06]**	0.89 [0.75, 1.04]***	0.32 [−0.04, 0.68]
Maternal employment during pregnancy	−1.54 [−2.01, −1.08]***	−0.29 [−1.34, 0.75]	−1.06 [−1.44, −0.68]***	−0.29 [−1.16, 0.59]	−0.48 [−0.61, −0.35]***	−0.01 [−0.27, 0.26]
Preterm birth (<37 weeks)	0.53 [−0.19, 1.25]	−0.68 [−2.16, 0.79]	0.43 [−0.16, 1.01]	−0.44 [−1.68, 0.79]	0.1 [−0.1, 0.31]	−0.24 [−0.61, 0.13]
Low birthweight (<2500 g)	0.66 [−0.14, 1.46]	−0.16 [−1.82, 1.5]	0.52 [−0.13, 1.17]	−0.07 [−1.46, 1.32]	0.14 [−0.09, 0.37]	−0.09 [−0.51, 0.32]
Breastfed at 12 months	0.16 [−0.32, 0.64]	0.52 [−0.55, 1.59]	0.08 [−0.31, 0.47]	0.54 [−0.35, 1.44]	0.08 [−0.06, 0.22]	−0.02 [−0.29, 0.25]
Neurocognitive development (BDI > 1.5 SD)	0.61 [−0.52, 1.74]	0.07 [−2.28, 2.43]	0.34 [−0.58, 1.26]	0.33 [−1.65, 2.31]	0.27 [−0.05, 0.6]	−0.25 [−0.85, 0.34]
**Family structure**						
Maternal age (years)	−1.39 [−1.79, −0.99]***	−0.96 [−1.92, 0]	−0.94 [−1.27, −0.62]***	−0.69 [−1.5, 0.12]	−0.45 [−0.56, −0.33]***	−0.27 [−0.51, −0.03]*
Parity	0.48 [0.21, 0.74]***	−0.17 [−0.83, 0.48]	0.37 [0.16, 0.59]***	−0.12 [−0.67, 0.43]	0.1 [0.03, 0.18]**	−0.06 [−0.22, 0.11]
Maternal marital status	1.6 [0.97, 2.22]***	1.48 [−0.04, 3.01]	1.19 [0.68, 1.7]***	1.07 [−0.21, 2.35]	0.4 [0.22, 0.58]***	0.42 [0.03, 0.8]
**Maternal mental health and parenting**						
Maternal depression (EPDS)	3.44 [2.92, 3.95]***	0.94 [−0.43, 2.31]	2.77 [2.36, 3.19]***	0.82 [−0.32, 1.97]	0.66 [0.51, 0.81]***	0.12 [−0.23, 0.46]
Harsh parenting (parent–child interactions)	3.07 [1.92, 4.22]***	4.56 [2.44, 6.68]***	2.02 [1.08, 2.96]***	4.02 [2.24, 5.8]***	1.05 [0.72, 1.38]***	0.54 [0.01, 1.07]
Child stimulation	−1.13 [−1.35, −0.91]***	−0.17 [−0.73, 0.38]	−0.77 [−0.95, −0.59]***	−0.17 [−0.63, 0.3]	−0.36 [−0.43, −0.3]***	−0.01 [−0.14, 0.13]

Unstandardised regression coefficients (*b*) which represent unadjusted associations, and corresponding 95% confidence intervals (CIs), between explanatory variables and child behavioural problems. *P* values adjusted for multiple testing using the Benjamini–Hochberg method to control for the false discovery rate. BDI, Battelle’s Development Inventory; CBCL, Child Behaviour Checklist; EPDS, Edinburgh Postnatal Depression Scale; SD, standard deviation.

* *P* < .05,

** *P* < .01,

*** *P* < .001.

Explanatory models that controlled for risk/protective factors continued to show a significant reduction in child behavioural problems between the 2004 and 2015 cohorts (Table 3). However, controlling for demographic, socioeconomic and risk/protective factors attenuated the reduction over time. For example, the effect of cohort was attenuated by 22%, from −2.77 (95% confidence interval [CI]: −3.38, −2.15; *P* < 0.001) to −2.16 (95% CI: −2.81, −1.52; *P* < 0.001), after controlling for change in perinatal health and developmental factors. This attenuation may be attributed to reduced maternal smoking and unemployment during pregnancy. In fully adjusted models, the cohort effect was attenuated even further to −1.69 (95% CI = −2.34, −1.05) for conduct problems (39% total attenuation), reflecting changes in social inequality, maternal characteristics (e.g., age, depression) and behaviours (e.g., smoking during pregnancy, parenting) that are related to child behavioural problems (Table S7).

**Table 3.** Change in child behavioural problems between the 2004 and 2015 cohorts (unadjusted and adjusted for change in explanatory variables)

	Conduct problems	Aggressive behaviour	Rule-breaking behaviour
**Model 1 (**unadjusted)	−2.77 [−3.38, −2.15]***	−1.97 [−2.48, −1.47]***	−0.79 [−0.97, −0.62]***
**Model 2** (adjusted, age)	−2.75 [−3.6, −1.9]***	−1.84 [−2.54, −1.15]***	−0.91 [−1.15, −0.67]***
**Model 3** (adjusted, demographic and socioeconomic factors)	−2.46 [−3.08, −1.85]***	−1.76 [−2.26, −1.26]***	−0.7 [−0.88, −0.53]***
**Model 4** (adjusted, prenatal and developmental factors)	−2.16 [−2.81, −1.52]***	−1.54 [−2.07, −1.01]***	−0.62 [−0.81, −0.44]***
**Model 5** (adjusted, family structure)	−2.23 [−2.86, −1.59]***	−1.6 [−2.12, −1.07]***	−0.63 [−0.81, −0.45]***
**Model 6** (adjusted, maternal mental health and parenting)	−2.31 [−2.92, −1.69]***	−1.63 [−2.13, −1.12]***	−0.68 [−0.86, −0.5]***
**Model 7** (fully adjusted, trimmed)	−1.69 [−2.34, −1.05]***	−1.27 [−1.8, −0.73]***	−0.74 [−0.99, −0.49]***

Statistics represent the main effects of cohort (*b*) and corresponding 95% confidence intervals on each CBCL outcome. Model 1 unadjusted; Model 2 adjusted for child’s age at time of testing; Model 3 adjusted for demographic and socioeconomic factors (child’s sex, ethnic background, family income and maternal education); Model 4 adjusted for prenatal and developmental factors (maternal smoking and employment during pregnancy, preterm birth, low birthweight, breastfed at 12 months and neurocognitive development); Model 5 adjusted for family structure (maternal age, parity and maternal marital status); Model 6 adjusting for maternal mental and parenting (maternal depression, harsh parenting and child stimulation); Model 7 fully adjusted for all explanatory variables retained in backward elimination models (Table S8). BDI, Battelle’s Development Inventory; CBCL, Child Behaviour Checklist; EPDS, Edinburgh Postnatal Depression Scale.

* *P* < .05,

** *P* < .01,

*** *P* < .001.

## Discussion

This epidemiological study compared three population-based cohorts over a 22-year period and found that a previously reported increase in conduct problems and aggression among 4-year-old Brazilian children from 1997 to 2008 (1993 vs 2004 cohort) was followed by a decline from 2008 to 2019 (2004 vs 2015 cohort). Rule-breaking behaviours also decreased from 2008 to 2019, and boys no longer showed higher levels of behavioural problems than girls by 2019. Despite overall recent reductions in behavioural problems, we find that the socioeconomic inequalities in behavioural problems that emerged between 1997 and 2008 have persisted. The reductions in child behavioural problems from 2008 to 2019 were partly, but not fully, explained by changes in social inequalities, prenatal and developmental factors, family structure, and maternal mental health and depression.

Our study updates and expands on a previous study that identified a rise in conduct problems and aggression in 4-year-olds from 1997 to 2008 in Pelotas, Brazil (Matijasevich *et al*., 2014). Here, we show the important and novel finding that this trend did not continue; rather behaviour problems then decreased from 2008 to 2019. This suggests that, like in high-income countries, secular trends may be levelling off and Brazil may be seeing similar – albeit delayed – population improvements in behavioural problems more recently (Collishaw, 2015; Maughan *et al*., 2008; Sellers *et al*., 2015). This delayed improvement mirrors national trends in crime. Although most high-income countries saw marked reductions in police- and victim-reported crime in the mid- to late-1990s, national crime trends in Brazil only began to fall in 2017 (Cerqueira *et al*., 2021; Murray *et al*., 2013). Tracking trends in child behavioural problems may be key to anticipating crime and violence at both the individual and population level (Loeber *et al*., 1993; Moffitt, 1993). With previous evidence showing that child behavioural problems predict subsequent crime and violence in Brazil (Murray *et al*., 2015), the recent decline in child behavioural problems may signal future improvements. Future research should aim to monitor whether these changes in early life translate into reduced future crime and violence at the population level.

Despite promising overall reductions in child behavioural problems, we find a concerning social gap in recent years. Since 2008, young children from poorer families and of less educated mothers are at increased risk of conduct problems and aggression. These findings echo concerns about evidence of widening disparities in child mental health problems in high-income countries (Collishaw *et al*., 2019; Collishaw and Sellers, 2020) and raise the critical question of why socially disadvantaged children have become more vulnerable with time (Melchior, 2021). While Brazil has made substantial progress in many social and health domains (e.g., life expectancy, child labour), our findings suggest that these advances might not be felt among all children equally (Marmot, 2016; Paim *et al*., 2011). One possible explanation is the ‘inverse equity hypothesis’, which posits that inequities often increase, despite overall improvements in health, since public health interventions initially reach those of higher socioeconomic status and only later affect the more disadvantaged (Victora *et al*., 2000). Since we found that the social gap in child behavioural problems has not deteriorated in recent years – only persisted – this might suggest a levelling off of differences as improvements in health begin to reach more disadvantaged children in Brazil. Pinpointing differences among socially advantaged and disadvantaged children, and how these relate to child mental health, is an important area of research in order to achieve mental health equity for all.

In contrast to the emergence of a social gap in child behavioural problems over the 22-year period, we found that sex differences in 1997 for rule-breaking behaviour disappeared by 2019. This is because there was a larger reduction in rule-breaking behaviour from 2008 to 2019 among boys compared to girls. We found no sex differences for aggression or total conduct problems during the entire study period. Previous evidence indicates clear sex differences where behavioural problems (particularly aggression) are more commonly reported in boys than girls (Lahey *et al*., 2006; Tiet *et al*., 2001). It is likely that we did not identify sex differences for aggression or total conduct problems as it is hypothesised that differences emerge around 4-years-old and widen during childhood (Keenan and Shaw, 1997). While it is unclear why boys are at higher risk of behavioural problems (Lahey *et al*., 2006), our finding that sex differences for rule-breaking behaviour were already reported at 4-years-old, yet did not persist across all cohorts, suggests that social changes and norms might be influencing the manifestation, perception and reporting of rule-breaking behaviour. This undermines a purely genetic explanation for sex differences in rule-breaking behaviour and evokes further questions concerning wider environmental and societal influences on early behaviour (Wood and Eagly, 2002).

### Strengths and limitations

This study compared three birth cohort studies that used comparable methods to sample from the same target population of all births in Pelotas, Brazil. The equivalence of the target population, sampling methodology, data collection procedures and measurement methods ensured that we were able to directly test for secular changes in behavioural problems among 4-year-olds over 22 years. In addition, the longitudinal data within cohorts allowed us to test for changes in social inequalities in child behavioural problems across the three cohorts and investigate the role of early risk and protective factors that may offer potential explanations of change between the two more recent cohorts.

The study also had several limitations. First, the 4-year assessment for the 1993 and 2015 cohorts were based on subsamples rather than the whole cohort. Although the 2015 cohort’s subsample was random and representative, the 1993 subsample oversampled low birthweight children. To address this issue, we applied sample-specific weights to ensure that the subsample represented all cohort children – as in previous research with this cohort (Barros *et al*., 2012; Matijasevich *et al*., 2014). Second, the cohorts represent children born in a single Brazilian southern middle-sized urban city. Our findings may not therefore generalise to children living elsewhere. Third, this study is unable to disentangle whether changes in behavioural problems were due to period or cohort effects, or a combination of the two (Collishaw, 2015). Finally, we rely on parent reports to measure child behavioural problems across the three cohorts and thus examine secular changes in the reporting of behavioural problems which may not necessarily reflect true changes in child behavioural problems. It is generally assumed that parents have become more willing to report problems as mental health stigma has reduced (Beers and Joshi, 2020), yet we find changes in the opposite direction. This suggests that changes in reporting bias alone do not account for the reductions between the 2004 and 2015 cohorts. In addition, socioeconomic factors, such as maternal education, might influence parent reports of child behavioural problems, accounting for some of the observed social gap in child behavioural problems in recent years. Although there is limited evidence that maternal education influences the reporting of child behavioural problems and it is unlikely that this potential measurement error has changed over time (Stone *et al*., 2013), future studies should aim to incorporate convergent measures across multiple methods and/or informants to triangulate such findings.

## Conclusions

This study updates the evidence on secular trends in child mental health problems in Brazil and finds that behavioural problems among young children have reduced since 2008. Critically, however, these improvements were not felt equally among all children. By 2008, there were marked differences in behavioural problems between socially advantaged and disadvantaged children, and these socioeconomic inequalities in behavioural problems have not ameliorated with time. Our findings thus highlight the importance of monitoring trends by subgroups in low- and middle-income countries to better understand, and respond to, the risks facing the most vulnerable children around the world. Closing the mental health gap between socially advantaged and disadvantaged children is a necessary public health priority to reduce cascading impacts on the next generation.

## Data Availability

Data from the studies ‘Pelotas Birth Cohort, 1993’, ‘Pelotas Birth Cohort, 2004’ and ‘Pelotas Birth Cohort, 2015’ are available by special request to Postgraduate Program in Epidemiology at Universidade Federal de Pelotas via the following website: http://www.epidemio-ufpel.org.br/site/content/studies/. MDE had full access to all of the data in the study and takes responsibility for the integrity of the data and the accuracy of the data analysis.

## Supplementary material

For supplementary material accompanying this paper visit http://doi.org/10.1017/S2045796023000185. click here to view supplementary material
