# Supplementary material for: Secular trends and social inequalities in child behavioural problems across three Brazilian cohort studies (1993, 2004 and 2015)
Source: Epidemiol Psychiatr Sci. 2023 Apr 17;32:e23. doi: 10.1017/S2045796023000185 (PMC10130841; doi:10.1017/S2045796023000185)
Supplement: Supplementary file 1 [file epssup.zip › S2045796023000185sup002.pdf]

Supporting Information

Secular trends and social inequalities in child behavioural problems across three Brazilian cohort studies (1993, 2004, and 2015) – by Degli Esposti *et al.*

Contents Page

Appendix

Appendix S1. Power calculations 2

Appendix S2. Inequality indices for family income and maternal education 3

Supplementary tables

Table S1. Measures of demographic and socioeconomic factors hypothesised to moderate changes in child behavioural problems 4

Table S2. Child behavioural problems across the 1993, 2004, and 2015 Pelotas birth cohorts 5

Table S3. Demographic and socioeconomic measures in the 1993, 2004 and 2015 Pelotas birth cohorts 6

Table S4. Social inequalities in child behavioural problems in the 1993, 2004, and 2015 Pelotas birth cohorts 7

Table S5. Indices of social inequality in child behavioural problems for family income and maternal education in the 1993, 2004, and 2015 Pelotas birth cohorts 8

Table S6. Risk and protective factors in the 2004 and 2015 Pelotas birth cohorts 9

Table S7. Fully adjusted, trimmed models explaining the reduction in child behavioural problems from the 2004 to 2015 cohort 10

Supplementary figures

Figure S1. Timeline of the 1993, 2004, 2015 Pelotas birth cohorts and their follow-ups. 11

Figure S2. Power calculations using simulated two-sample t-tests to estimate minimum sample sizes for 80% power 12

Figure S3. Power analyses for regression models where the interaction the term of interest (e.g., income x cohort), also known as moderation analyses. 13

Figure S4. Schematic diagram (DAG) showing the hypothesised associations between the explanatory variables and child behavioural problems, and how differences in these variables might explain changes over time. 14

Figure S5. Directed acyclic graph (DAG) comparing unadjusted and adjusted model estimates to explore whether changes in child's age, social inequalities, prenatal (infant) and developmental factors, family structure, and maternal mental health and parenting explain changes in child behavioural problems between the 2004 and 2015 cohort. 15

Supplemental references 16

Appendix S1. Power calculations

This study is an analysis of three cohort studies post data collection, and thus sample sizes are already determined (see methods in manuscript). However, to ensure our main and stratified analyses can detect significance at the statistical and clinical level, we conducted power calculations. Specifically, we used simulations which were informed by results from a previous study in the same population to identify minimum sample sizes (Matijasevich *et al.*, 2014).

We investigated power for detecting differences in child behavioural problems across cohorts by simulating a series of two sample t-tests using previous Cohen's d estimates for CBCL scores for total externalising problems and aggressive behaviour (Matijasevich *et al.*, 2014). The results are shown in Figure S2 and indicate that sample sizes of 100 are needed for each group to detect differences in mean CBCL scores across cohorts with >80% power, when adopting the more conservative Cohen's d estimate of 0.40. For our main analysis, we adopted a conservative approach and used our smallest possible sample sizes for each cohort (n=600) in follow-up power calculations. Here, we identified that with our sample sizes we will be able to detect a statistically significant ($p<0.05$) small difference (Cohen's d = 0.16) between cohorts with >80% power.

For our moderation analyses, we used conservative estimates for our pooled sample size across all three cohorts (n=4000) to test power in detecting interaction effects in regression models. To inform these power analyses, we drew from the aforementioned Brazilian study (Matijasevich *et al.*, 2014) and a UK study (Collishaw *et al.*, 2019) that specifically investigated interaction effects between income and trends in child mental health trends across three cohorts. These studies identified small yet significant correlations between income, child mental health, and cohort. We therefore used a Pearson's correlation of 0.1 for the associations between all three variables. We then varied the effect size of an interaction effect (i.e., income x cohort on child mental health) by 0.01 intervals from 0.01 to 0.1. Figure S3 shows that, with >80% power, we will be able to detect an interaction effect size of 0.045 and over when using a sample size of 4000.

Appendix S2. Inequality indices for family income and maternal education

Our main models used dichotomised measures of family income and maternal education, in line with our power calculations (see Appendix S1 and Table S1). In order to also model the whole distribution of family income and maternal age, we derived two inequality indices to measure social inequalities in child behavioural problems (Harper and Lynch, 2005; Barros and Victora, 2013). Specifically, we calculated the slope index of inequality (SII) and relative index of inequality (RII) in relation to child behavioural problems. The SII is the absolute difference in CBCL scores between children in the highest (e.g., richest) and lowest (e.g., poorest) socioeconomic position, whereas RII is the ratio of estimated CBCL scores between a child in the lowest socioeconomic position and a child in the highest socioeconomic position (Kunst and Mackenbach, 1990). To derive the SII and RII, we calculated rank scores (or relative ranks), which account for differences in sample sizes in each subgroup as they are based on the midpoint of its range in the cumulative distribution of the total sample size. Rank scores were then modelled as predicting CBCL scores, and the resulting regression line represented the SII.

Table S1. Measures of demographic and socioeconomic factors hypothesised to moderate changes in child behavioural problems

Measure	Description	Child's age at measurement	Response categories	Collapsed response categories
**Demographic**				
Sex	Child's sex	Birth	2 categories: male vs female	n/a
Ethnic background[a]	Mother's skin colour: White, and Black/mixed (including Brown with mixed European and African ancestry)	Birth	2 categories: White vs Black/mixed	n/a
**Socioeconomic position**				
Family income	Based on the family's household income	Birth	5 categories: quintiles	2 categories: poorest (Q1-Q2) and richest (Q3-Q5)
Maternal education	The number of years of the mother completed of schooling	Birth	3 categories: 0-4, 5-8, and ≥9y	2 categories: low (0-8y) vs high (≥9y)

Q, quintiles; y, years.
[a]Based on previous literature (Matijasevich *et al.*, 2014).
[b]We collapsed categories subgroup sample sizes are below n=100 (see Appendix S1 for power calculations).

Table S2. Child behavioural problems across the 1993, 2004, and 2015 Pelotas birth cohorts

	CBCL score, Mean (SEM)			Cohort comparisons, *b* [95% CI]		
	**1993 cohort**	**2004 cohort**	**2015 cohort**	**1993 vs 2004 cohort**	**2004 vs 2015 cohort**	**1993 vs 2015 cohort**
**Unadjusted**						
Conduct problems	12.24 (0.36)	15.48 (0.12)	12.72 (0.26)	3.24 [2.51, 3.98]***	-2.77 [-3.33, -2.2]***	0.48 [-0.39, 1.35]
Aggressive behaviour	9.85 (0.29)	12.95 (0.1)	10.98 (0.22)	3.11 [2.51, 3.7]***	-1.97 [-2.45, -1.5]***	1.13 [0.42, 1.85]**
Rule-breaking behaviour	2.39 (0.1)	2.53 (0.03)	1.73 (0.07)	0.14 [-0.07, 0.34]	-0.79 [-0.94, -0.65]***	-0.66 [-0.89, -0.42]***
**Adjusted for child's age (months) at time of testing**						
Conduct problems	11.6 (3.01)	15.35 (2.83)	12.11 (3.5)	3.28 [2.5, 4.06]***	-2.75 [-3.56, -1.94]***	0.59 [-0.87, 2.05]
Aggressive behaviour	7.96 (2.41)	11.7 (2.34)	9.77 (2.79)	3.22 [2.58, 3.85]***	-1.84 [-2.51, -1.17]***	1.36 [0.17, 2.54]*
Rule-breaking behaviour	3.64 (0.88)	3.65 (0.78)	2.34 (1.02)	0.06 [-0.16, 0.28]	-0.91 [-1.13, -0.69]***	-0.77 [-1.18, -0.35]***

Unstandardised regression coefficients (*b*) represent mean differences in CBCL scores between cohorts.
CBCL, Child Behaviour Checklist; CI; confidence intervals; SEM, standard error of mean.
**p* < .05; ***p* < .01; ****p* < .001.

Table S3. Demographic and socioeconomic measures in the 1993, 2004 and 2015 Pelotas birth cohorts

**Characteristic**	**1993 cohort (N = 633)**	**2004 cohort (N = 3,750)**	**2015 cohort (N = 577)**
Child's age at time of testing	53.6 (3.7)	50.3 (1.8)	45.0 (2.5)
Child's sex			
Male	49.5% (312.8)	51.8% (1,943)	53.9% (311)
Female	50.5% (318.8)	48.2% (1,807)	46.1% (266)
Ethnic background (mother's skin color)			
White	77.1% (487.1)	73.1% (2,741)	69.5% (401)
Black/mixed	22.9% (144.5)	26.9% (1,009)	30.5% (176)
Family income (quintiles)			
Poorest	18.1% (112.7)	19.9% (748)	16.3% (94)
2nd	23.2% (144.8)	20.1% (753)	24.3% (140)
3rd	21.0% (130.6)	19.9% (745)	21.7% (125)
4th	17.7% (110.1)	20.8% (779)	18.7% (108)
Richest	20.0% (124.7)	19.3% (725)	19.1% (110)
Maternal education (schooling years)			
0-4	27.3% (171.9)	15.1% (560)	7.6% (44)
5-8	45.2% (285.0)	41.7% (1,550)	26.2% (151)
≥9	27.5% (173.1)	43.2% (1,603)	66.2% (382)

Percentages (sample sizes) are presented for categorial variables while mean (standard deviation) is presented for the continuous variables of child's age at time of testing at 4-year assessment. The number of children in the 1993 cohort are not integers due to weighting adjustment to account for oversampling of low birthweight children.

Table S4. Social inequalities in child behavioural problems in the 1993, 2004, and 2015 Pelotas birth cohorts

	Mean CBCL score (SD)								
	**Conduct problems**			**Aggressive behaviour**			**Rule-breaking behaviour**		
	**1993 cohort**	**2004 cohort**	**2015 cohort**	**1993 cohort**	**2004 cohort**	**2015 cohort**	**1993 cohort**	**2004 cohort**	**2015 cohort**
**Child's sex**									
Male	12.70 (7.97)	15.70 (7.44)	12.64 (6.47)	10.02 (6.36)	13.00 (6.04)	10.87 (5.41)	2.68 (2.49)	2.70 (2.15)	1.77 (1.62)
Female	11.79 (8.43)	15.25 (6.81)	12.80 (6.20)	9.68 (6.97)	12.91 (5.60)	11.11 (5.21)	2.11 (2.10)	2.34 (1.93)	1.69 (1.56)
Mean difference, *b*	-0.91	-0.46	0.16	-0.35	-0.09	0.24	-0.57	-0.36	-0.08
[95% CI]	[-2.31, 0.48]	[-0.91, 0]	[-0.88, 1.19]	[-1.48, 0.78]	[-0.46, 0.28]	[-0.63, 1.11]	[-0.96, -0.17]**	[-0.49, -0.23]***	[-0.34, 0.18]
**Ethnic background (mother's skin color)**									
White	12.26 (8.14)	15.02 (6.99)	12.40 (6.10)	9.84 (6.64)	12.62 (5.75)	10.74 (5.19)	2.42 (2.32)	2.40 (1.96)	1.66 (1.47)
Black/mixed	12.18 (8.48)	16.75 (7.42)	13.44 (6.81)	9.87 (6.79)	13.86 (5.94)	11.53 (5.57)	2.31 (2.30)	2.88 (2.26)	1.91 (1.82)
Mean difference, *b*	-0.08	1.73	1.05	0.03	1.24	0.8	-0.11	0.49	0.25
[95% CI]	[-1.79, 1.62]	[1.2, 2.26]***	[-0.12, 2.22]	[-1.34, 1.39]	[0.82, 1.67]***	[-0.17, 1.76]	[-0.58, 0.36]	[0.33, 0.65]***	[-0.05-0.56]
**Family income**									
Richer (Q3-Q5)	12.23 (7.90)	14.54 (6.69)	11.96 (5.68)	9.84 (6.53)	12.30 (5.56)	10.37 (4.85)	2.38 (2.10)	2.24 (1.85)	1.59 (1.42)
Poorer (Q1-Q2)	12.16 (8.57)	16.89 (7.57)	13.82 (7.07)	9.78 (6.84)	13.93 (6.07)	11.87 (5.83)	2.38 (2.57)	2.96 (2.25)	1.95 (1.80)
Mean difference, *b*	-0.07	2.35	1.86	-0.06	1.63	1.5	0	0.72	0.36
[95% CI]	[-1.51, 1.38]	[1.88, 2.82]***	[0.77, 2.94]***	[-1.22, 1.1]	[1.25, 2.01]***	[0.59, 2.4]**	[-0.42, 0.42]	[0.58, 0.86]***	[0.09, 0.63]*
**Maternal education (schooling years)**									
Higher (≥9y)	12.18 (7.28)	13.94 (6.29)	12.30 (5.79)	9.67 (6.10)	11.88 (5.31)	10.68 (4.92)	2.51 (2.06)	2.06 (1.68)	1.62 (1.46)
Lower (0-8y)	12.28 (8.54)	16.63 (7.55)	13.53 (7.25)	9.93 (6.88)	13.74 (6.08)	11.57 (5.99)	2.35 (2.41)	2.89 (2.23)	1.96 (1.80)
Mean difference, *b*	0.1	2.7	1.23	0.26	1.86	0.9	-0.16	0.84	0.34
[95% CI]	[-1.37, 1.57]	[2.25, 3.14]***	[0.07, 2.4]*	[-0.95, 1.47]	[1.49, 2.23]***	[-0.08, 1.87]	[-0.57, 0.26]	[0.71, 0.96]***	[0.05, 0.63]*

Subgroups with sample sizes of less than 100 were collapsed as per power calculations [see Appendix S1 and Figures S2-S3). Mean difference was estimated using separate [unadjusted) linear regressions with dichotomised demographic and socioeconomic measures entered as predictors. *b;* beta coefficient; CBCL, Child Behaviour Checklist; CI; confidence intervals; Q, quintiles; SD; standard deviation; y, years.
**p* < .05; ***p* < .01; ****p* < .001.

Table S5. Indices of socioeconomic inequality in child behavioural problems for family income and maternal education in the 1993, 2004, and 2015 Pelotas birth cohorts

Socioeconomic position measure	Mean CBCL score (SD)								
	Conduct problems			Aggressive behaviour			Rule-breaking behaviour		
	1993 cohort	2004 cohort	2015 cohort	1993 cohort	2004 cohort	2015 cohort	1993 cohort	2004 cohort	2015 cohort
**Family income (quintiles)**									
Poorest	12.19 (8.06)	16.93 (7.42)	14.44 (7.71)	9.70 (6.69)	13.96 (5.97)	12.41 (6.44)	2.49 (2.29)	2.97 (2.23)	2.02 (1.82)
2nd	12.14 (8.98)	16.85 (7.73)	13.41 (6.60)	9.85 (6.97)	13.91 (6.18)	11.51 (5.38)	2.30 (2.77)	2.95 (2.28)	1.90 (1.78)
3rd	12.30 (7.98)	15.97 (7.18)	12.40 (5.85)	10.10 (6.50)	13.41 (5.95)	10.71 (4.87)	2.20 (2.09)	2.57 (1.99)	1.69 (1.58)
4th	12.30 (8.04)	14.40 (6.49)	11.94 (5.71)	9.56 (6.51)	12.17 (5.44)	10.25 (4.96)	2.75 (2.17)	2.24 (1.77)	1.69 (1.39)
Richest	12.09 (7.75)	13.22 (6.07)	11.49 (5.47)	9.83 (6.61)	11.31 (5.07)	10.11 (4.74)	2.26 (2.03)	1.90 (1.73)	1.38 (1.24)
SII	-0.01	-4.91	-3.51	0	-3.5	-2.79	-0.01	-1.41	-0.72
[95% CI]	[-2.44, 2.42]	[-5.69, -4.13]***	[-5.39, -1.63]***	[-2, 2]	[-4.13, -2.87]***	[-4.38, -1.2]***	[-0.7, 0.68]	[-1.65, -1.17]***	[-1.17, -0.27]**
RII	1	1.38	1.32	1	1.31	1.29	1	1.77	1.52
**Maternal education (schooling years)**									
0-4y	13.81 (9.56)	17.09 (8.09)	14.70 (8.77)	11.10 (7.41)	14.04 (6.38)	12.80 (7.37)	2.72 (2.95)	3.05 (2.46)	1.91 (1.83)
5-8y	11.36 (7.74)	16.47 (7.34)	13.19 (6.74)	9.23 (6.45)	13.63 (5.96)	11.22 (5.51)	2.13 (1.98)	2.84 (2.14)	1.97 (1.80)
≥9y	12.18 (7.28)	13.94 (6.29)	12.30 (5.79)	9.67 (6.10)	11.88 (5.31)	10.68 (4.92)	2.51 (2.06)	2.06 (1.68)	1.62 (1.46)
SII	-2.24	-4.99	-2.66	-1.95	-3.43	-2.01	-0.29	-1.56	-0.65
[95% CI]	[-4.94, 0.46]	[-5.85, -4.13]***	[-5.07, -0.25]*	[-4.11, 0.21]	[-4.14, -2.72]***	[-4.03, 0.01]	[-1.11, 0.53]	[-1.81, -1.31]***	[-1.22, -0.08]*
RII	1.2	1.38	1.23	1.22	1.31	1.2	1.13	1.89	1.46

SII represents the absolute difference in CBCL scores between the top (e.g., richest) and bottom (e.g., poorest) social group, whereas RII represents the top:bottom social group ratio. CBCL, Child Behaviour Checklist; CI, confidence interval; RII, relative index of inequality; SD, standard deviation; SII, slope index of inequality; y, years.
*$p < .05$; **$p < .01$; ***$p < .001$.

Table S6. Risk and protective factors in the 2004 and 2015 Pelotas birth cohorts

	Characteristic	2004 cohort (N = 3,750)	2015 cohort (N = 577)	*p*-value
Demographic & socioeconomic factors	Child's sex			0.4
	Male	51.8% (1,943)	53.9% (311)	
	Female	48.2% (1,807)	46.1% (266)	
	Ethnic background (mother's skin color)			0.071
	White	73.1% (2,741)	69.5% (401)	
	Black/mixed	26.9% (1,009)	30.5% (176)	
	Family income (quintiles)			0.049
	Poorest	19.9% (748)	16.3% (94)	
	2nd	20.1% (753)	24.3% (140)	
	3rd	19.9% (745)	21.7% (125)	
	4th	20.8% (779)	18.7% (108)	
	Richest	19.3% (725)	19.1% (110)	
	Maternal education (schooling years)			<0.001
	0-4	15.1% (560)	7.6% (44)	
	5-8	41.7% (1,550)	26.2% (151)	
	≥9	43.2% (1,603)	66.2% (382)	
Prenatal & developmental factors	Maternal smoking during pregnancy			<0.001
	No	72.8% (2,731)	84.7% (487)	
	Yes	27.2% (1,019)	15.3% (88)	
	Maternal employment during pregnancy			<0.001
	No	59.3% (2,222)	43.7% (252)	
	Yes	40.7% (1,527)	56.3% (325)	
	Preterm birth (<37 weeks)			0.054
	No	88.4% (3,296)	85.6% (494)	
	Yes	11.6% (432)	14.4% (83)	
	Low birthweight (<2500 g)			0.2
	No	90.9% (3,409)	89.1% (514)	
	Yes	9.1% (340)	10.9% (63)	
	Breastfed at 12 months			0.10
	No	62.5% (2,286)	58.9% (330)	
	Yes	37.5% (1,371)	41.1% (230)	
	Neurocognitive development (BDI<1.5SD)			0.2
	Normal	95.7% (3,574)	94.5% (495)	
	Suspected delay	4.3% (161)	5.5% (29)	
Family structure	Maternal age (years)			<0.001
	≤19	18.8% (703)	10.6% (59)	
	20-34	67.5% (2,530)	69.0% (383)	
	≥35	13.7% (515)	20.4% (113)	
	Parity			<0.001
	0	39.4% (1,476)	49.8% (287)	
	1	26.6% (997)	29.3% (169)	
	≥2	34.0% (1,276)	20.8% (120)	
	Maternal marital status			0.12
	With partner	83.6% (3,057)	86.2% (482)	
	Single mother	16.4% (599)	13.8% (77)	
Maternal mental health & parenting	Maternal depression (EPDS)			<0.001
	No	72.8% (2,616)	81.9% (462)	
	Yes	27.2% (976)	18.1% (102)	
	Harsh parenting (parent-child interactions)			0.013
	No	95.9% (3,569)	93.6% (525)	
	Yes	4.1% (153)	6.4% (36)	
	Child stimulation	3.81 (1.03)	4.02 (0.94)	<0.001

Percentages (sample sizes) are presented for categorial variables while means (standard deviations) are presented for continuous variables. Absolute numbers do not always add to total number of participants due to missing data (all <5% missingness). BDI, Battelle's Development Inventory; EPDS, Edinburgh Postnatal Depression Scale; SD, standard deviation.

Table S7. Fully adjusted, trimmed models explaining the reduction in child behavioural problems from the 2004 to 2015 cohort.

	Conduct problems	Aggressive behaviour	Rule-breaking behaviour
**Cohort (2004 vs 2015 cohort)**	**-1.69 [-2.34, -1.05]*****	**-1.27 [-1.8, -0.73]*****	**-0.74 [-0.99, -0.49]*****
Child's age at time of testing	..		-0.04 [-0.07, -0.01]**
**Demographic & socioeconomic factors**			
Child's sex	-0.43 [-0.84, -0.01]*	..	-0.33 [-0.45, -0.21]***
Ethnic background (mother's skin colour)	0.77 [0.29, 1.25]**	0.58 [0.19, 0.97]**	0.2 [0.07, 0.34]**
Family income (quintiles)	-0.41 [-0.58, -0.24]***	-0.33 [-0.46, -0.19]***	-0.1 [-0.15, -0.05]***
Maternal education (schooling years)	-0.37 [-0.72, -0.03]*	..	-0.19 [-0.29, -0.09]***
**Prenatal & developmental factors**			
Maternal smoking during pregnancy	2.3 [1.81, 2.8]***	1.72 [1.31, 2.12]***	0.6 [0.46, 0.74]***
Maternal employment during pregnancy	..	..	..
Preterm birth (<37 weeks)	..	..	..
Low birthweight (<2500 g)	..	..	..
Breastfed at 12 months	..	..	..
Neurocognitive development (BDI>1.5D)	..	..	-0.21 [-0.51, 0.08]
**Family structure**			
Maternal age (years)	-1.03 [-1.4, -0.66]***	-0.69 [-1, -0.39]***	-0.33 [-0.43, -0.22]***
Parity	..	..	..
Maternal marital status	..	..	..
**Maternal mental health & parenting**			
Maternal depression (EPDS)	2.42 [1.94, 2.9]***	2.06 [1.66, 2.46]***	0.37 [0.24, 0.51]***
Harsh parenting (parent-child interactions)	..	1.25 [0.38, 2.11]**	0.54 [0.24, 0.84]***
Child stimulation	-0.5 [-0.72, -0.28]***	-0.33 [-0.51, -0.15]***	-0.18 [-0.24, -0.12]***

Unstandardised regression coefficients (*b*) which represent adjusted associations, and corresponding 95% confidence intervals, between explanatory variables and child behavioural problems. Explanatory variables with coefficients represent those which were retained as important for explaining change in child behavioural problems in the fully adjusted trimmed models using backward elimination. P values adjusted for multiple testing using the Benjamini-Hochberg method to control for the false discovery rate. BDI, Battelle's Development Inventory; CBCL, Child Behaviour Checklist; EPDS, Edinburgh Postnatal Depression Scale; SD, standard deviation.
**p* < .05; ***p* < .01; ****p* < .001.

PELOTAS BIRTH COHORT	**1993**	1994	1995	1996	1997	**2004**	2005	2006	2007	2008	2010	2011	2014-2015	**2015**	2016	2017	2018	2019
**1993** (n = 5,249)	**0**	1			**4**	11				15		18			23			
**2004** (n = 4,231)						**0**	1	2		**4**	6			11				15
**2015** (n = 4,275)													gestation	**0**	1	2		**4**

Figure S1. Timeline of the 1993, 2004, 2015 Pelotas birth cohorts and their follow-ups.
Note that bolded numbers represent key time-points for this study, while years coloured in grey represent follow-ups that fall outside the study period.

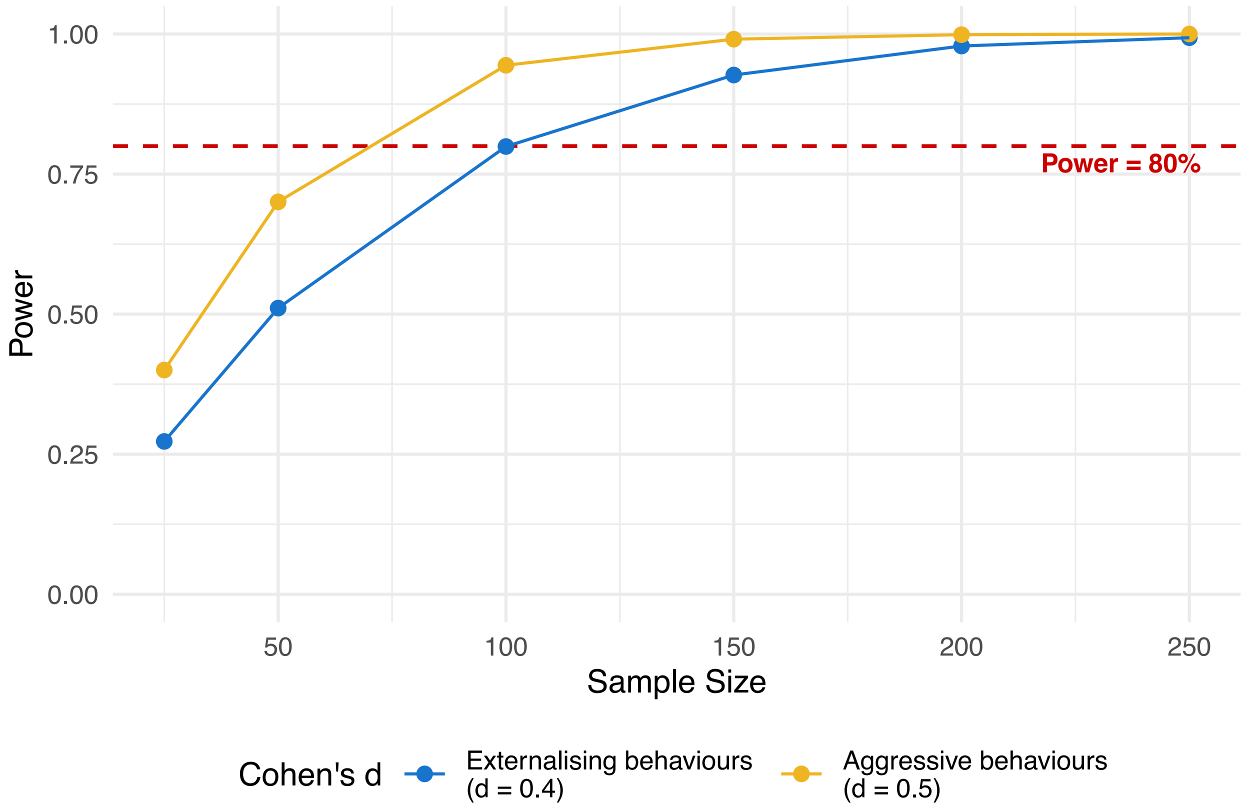

Figure S2. Power calculations using simulated two-sample t-tests to estimate minimum sample sizes for 80% power.

Note that Cohen's d values are informed by a previous study (Matijasevich *et al.*, 2014), where the estimated difference between the 1993 vs 2004 Pelotas cohorts were: d=0.4 for externalising behaviours and d=0.5 for aggressive behaviours.

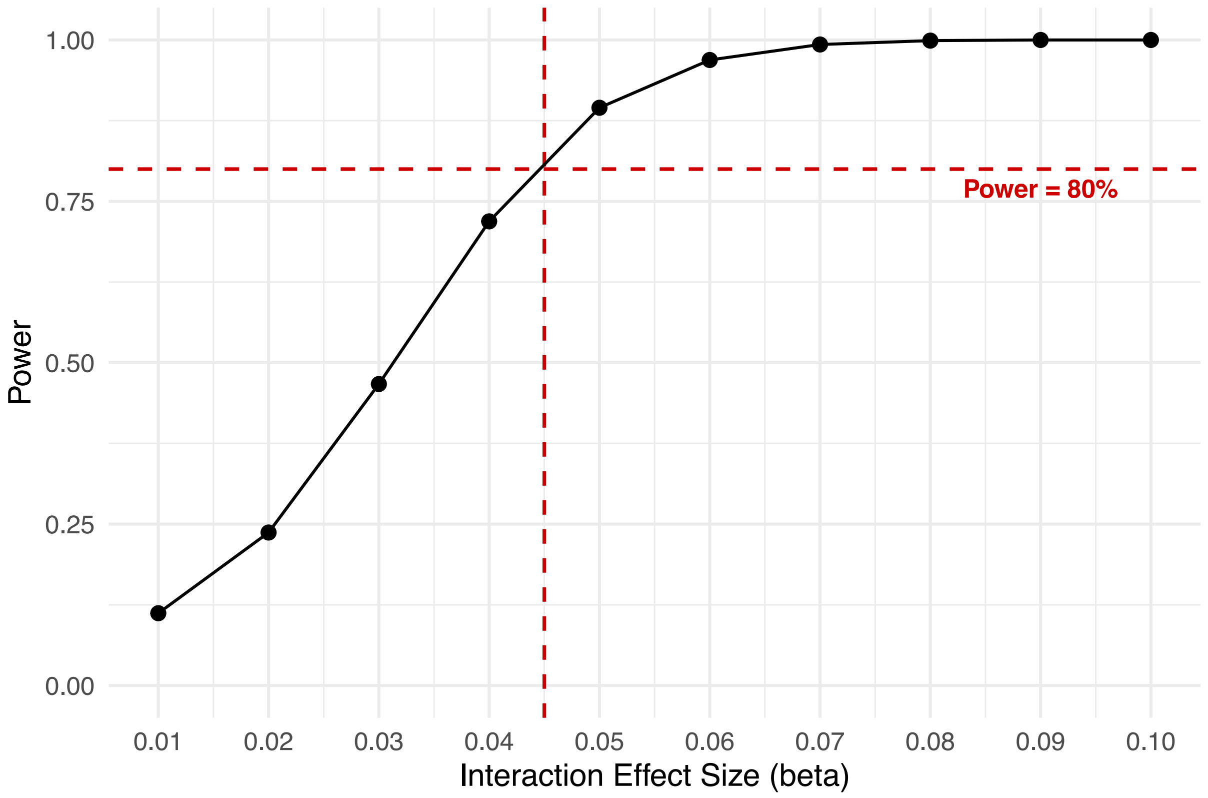

Figure S3. Power analyses for regression models where the interaction term is of interest (e.g., income x cohort), also known as moderation analyses.
Note that Pearson correlation values and the range of the interaction effect size is informed by a previous Brazilian (Matijasevich *et al.*, 2014) and UK study (Collishaw *et al.*, 2019).

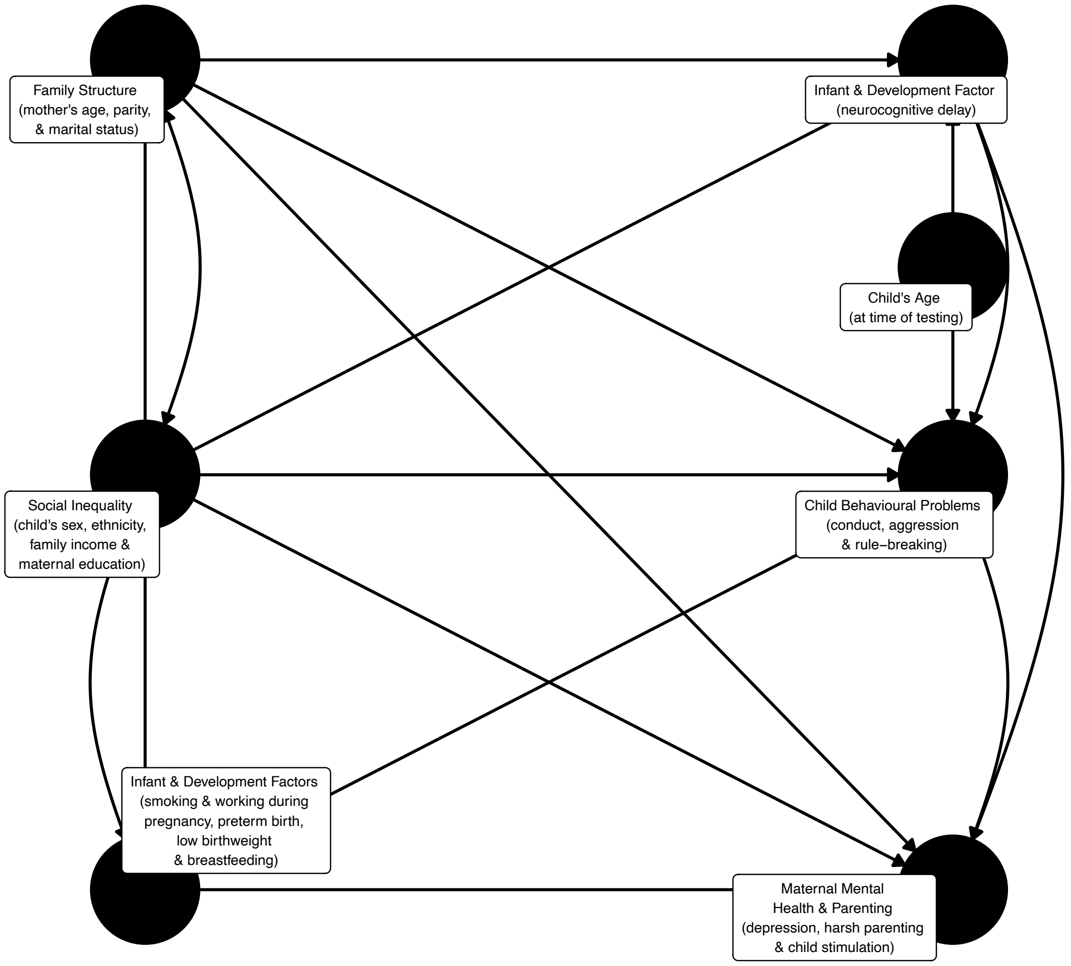

Figure S4. Schematic diagram (DAG) showing the hypothesised associations between the explanatory variables and child behavioural problems, and how differences in these variables might explain changes over time.

Note the potential of reverse causality between parenting, neurocognitive delay, and child behavioural problems as measures were collected during the same follow-up.

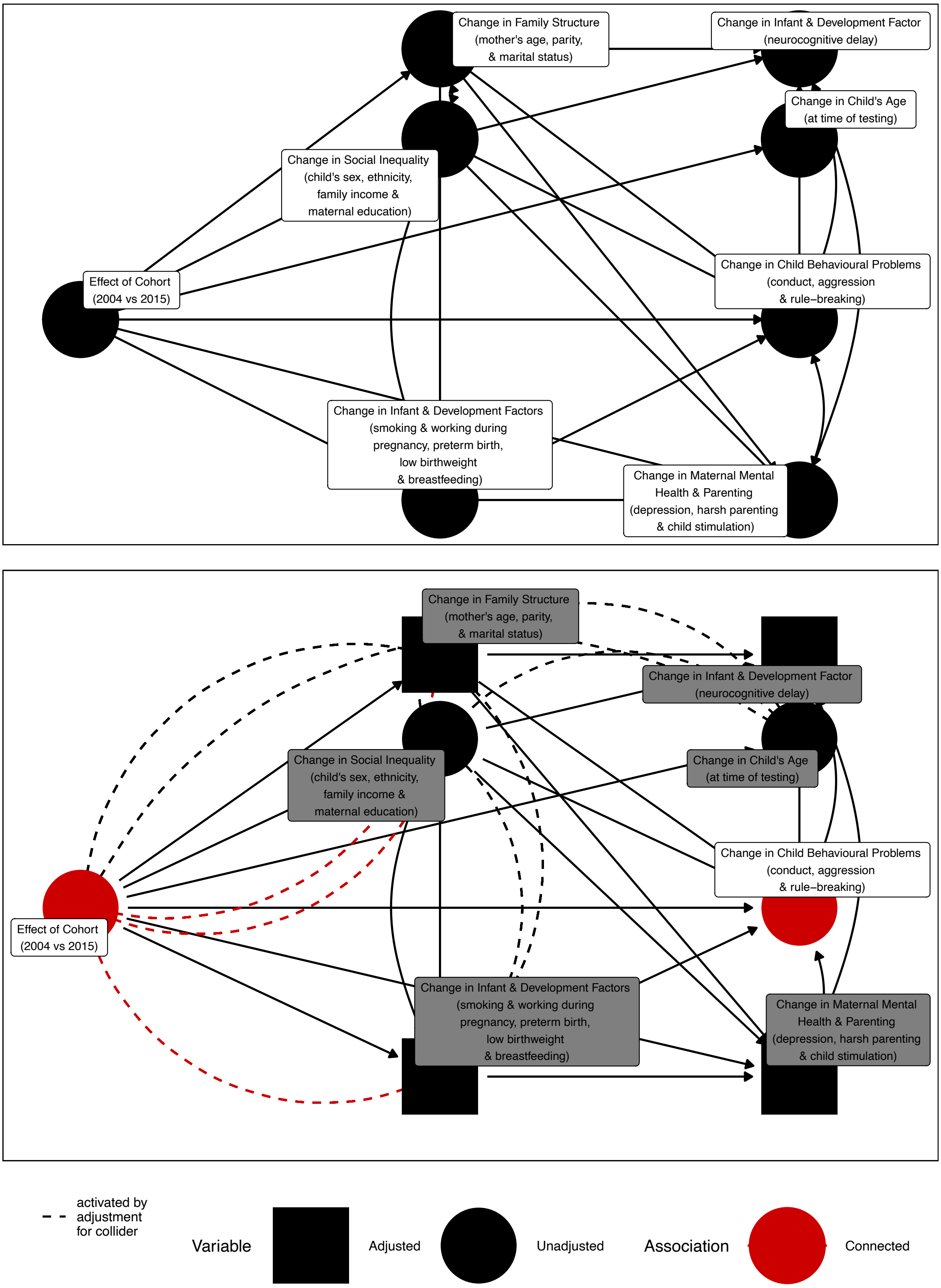

Figure S5. Directed acyclic graph (DAG) comparing unadjusted and adjusted model estimates to explore whether changes in child's age, social inequalities, prenatal (infant) and developmental factors, family structure, and maternal mental health and parenting explain changes in child behavioural problems between the 2004 and 2015 cohort.

Supplemental references

Barros, AJD and Victora, CG (2013) Measuring Coverage in MNCH: Determining and Interpreting Inequalities in Coverage of Maternal, Newborn, and Child Health Interventions. *PLOS Medicine* 10(5), e1001390. doi:10.1371/journal.pmed.1001390.

Collishaw, S, Furzer, E, Thapar, AK and Sellers, R (2019) Brief report: a comparison of child mental health inequalities in three UK population cohorts. *European Child & Adolescent Psychiatry* 28(11), 1547–1549. doi:10.1007/s00787-019-01305-9.

Harper, S and Lynch, J (2005) *Methods for measuring cancer disparities: using data relevant to healthy people 2010 cancer-related objectives*.

Kunst, AE and Mackenbach, JP (1990) Measuring socioeconomic inequalities in health, in *Measuring socioeconomic inequalities in health*, 115–115.

Matijasevich, A, Murray, E, Stein, A, Anselmi, L, Menezes, AM, Santos, IS, Barros, AJD, Gigante, DP, Barros, FC and Victora, CG (2014) Increase in child behavior problems among urban Brazilian 4-year olds: 1993 and 2004 Pelotas birth cohorts. *Journal of Child Psychology and Psychiatry* 55(10), 1125–1134. doi:10.1111/jcpp.12236.
